# How to get published in a standard peer-reviewed medical journal: Some useful tips for novice authors

**DOI:** 10.12669/pjms.36.6.3135

**Published:** 2020

**Authors:** Anbreen Aziz

**Keywords:** Scientific Writing

## Abstract

Young researchers and novice authors face lot of difficulties to document their research work and get it published due to lack of guidance and proper training in the art of scientific writing. This manuscript provides some important information and highlights some useful tips for novice authors which if followed in letter and spirit will minimize the trauma to their manuscripts with increased chances of publication in standard peer reviewed biomedical journals even those with Impact Factor. Other authors will also find it helpful to know the details related to the whole publishing process.

I had an enormous craving to write a brief communication after reading a manuscript “Publication Audit” and “editorials” recently published in Pakistan Journal of Medical Sciences regarding “common reasons for not accepting manuscripts for further processing after editor’s triage and initial screening” and “challenges faced by medical editors”.^1^-^3^ Since the human has handed over things to computer, revolution in the field of information technology has emerged with a shift from traditional to electronic publishing (e-publishing). Although e-publishing facilitates the authors, reviewers, and editors by trimming the publication process, it does create e-problems for the editors with the manuscript management softwares.^4^

Jawaid SA et al. in one of the editorials published in Pakistan Journal of Medical Sciences (PJMS) stated that number of submissions on the journal website has rapidly increased because, at present, only three biomedical journals enjoy impact factor including Pakistan Journal of Medical Sciences (PJMS), Journal of Pakistan Medical Association (JPMA) and Journal of College of Physicians & Surgeons Pakistan (JCPSP). They are covered by Clarivate Analytics USA Web of Sciences. The Journal Citation Report issued by Clarivate Analytics on June 30^th^ 2020, shows the current Impact Factor of these three journals 5 (Table-I)

**Table-I:** Current Impact Factor of Pakistani Medical Journals.

No.	Journal Name	Journal Impact Factor
1	Pakistan Journal of Medical Sciences	0.754
2	Journal of the Pakistan Medical Association	0.573
3	JCPSP - Journal of the College of Physicians and Surgeons Pakistan	0.426

Manuscripts are labelled as *“not accepted for further processing”* if rejected during initial screening before external peer review.^4^ PJMS gave detailed statistics of 2019 in which only 325 articles were published out of a total of 1419 submissions, while 981 were rejected.^1^ Some of the reasons for rejection of manuscripts (70-80%) at initial stages were:^2^

1. Failure to follow author’s instructions on journal website.
2. Lengthy manuscripts.
3. Poor English and Grammar.
4. Letter of undertaking not signed by all listed authors.
5. Too many authors with strong suspicion of gift authorship.
6. Poor literature search resulting in old references.
7. Too many submissions for journal to handle due to human resource and financial constraints.
8. Failure to provide Ethics Committee/IRB approval of study protocol.
9. Editor’s bias in choosing manuscripts with interesting topics.

It is well known that authors are impatient in getting their manuscripts published as early as possible hence, they are labelled as the most dangerous pressure group for the editors.^3^,^4^ Shaukat Ali Jawaid et al. in one of the editorials emphasized that editors have lot of tasks to do such as:^4^

1. Managing authors who are unfamiliar with online submission system.
2. Initial screening.
3. Formatting of accepted manuscripts.
4. Managing external review (sending online or printed copies according to reviewer’s choice).
5. Reviewing the reviewer’s comments to edit them to tone down harsh comments and omitting comments made for the editors themselves.
6. Improvement in English language and grammar.
7. Analyzing the manuscript along with response from corresponding author to reviewer’s comments to take decision of acceptance or rejection.
8. Final editing process and responsibility for the quality of manuscript accepted for publication.
9. Managing corrections in the pdf file a head of publication if authors do not know how to do it.
10. Finally managing left over corrections before printing the issue.

Editors are under constant pressure of increased workload and they do need sympathy and encouragement instead of harsh remarks and comments from authors. Cutting long story short, it is not an easy job for editors to make authors, reviewers, and readers happy owing to multiple task performance.^4^

Keeping in mind the whole publication process and editor’s workload, being novice author myself, I want other novice authors to benefit from few essential tips which I am sharing here:

1. Make a draft version of your study using conventional headings (Structured Abstract which should have sub-headings like objective, Methods, Results, Conclusions and appropriate Key words. The main text should have sections like Introduction, Methods, Results, Discussion and Conclusion, References).
2. If you are writing an article from your thesis, take a detailed review of your supervisor on that draft to correct it according to the instructions of your supervisor and keep making changes and taking feedbacks till the major issues are sorted out.
3. Once the draft is ready, search for journals according to your preferences. Search four to five preferred journals and send them emails regarding your intention of submission with the title of your study or you can even share the abstract. Few journals allow authors to first share the abstract on email to get an opinion about processing of the manuscripts.
4. Wait for few working days and if any journal responds to your query or asks you to submit, it simply means it is an active journal and you can send your manuscript there. This tip is important for all the novice authors.
5. Decide now who will be the corresponding author as he/ she should be familiar with e-journal system, scope and aim of journal with all other requirements such as word count and formatting.
6. Considering you the corresponding author, read few recently published articles of the targeted journal along with instructions for authors given on the journal website and modify your manuscript accordingly.
7. Next step is the submission phase which needs corresponding author to get registered on the journal’s website. Usually, the corresponding author needs to provide the following supplementary files along with main manuscript:
  - *Covering Letter*; Addressing to editor, mention title along with significance of the study.
  - *Authorship Letter*: It is usually provided by the journal and one needs to fill it up following the ICMJE (International Committee of Medical Journal Editors/Vancouver Group) authorship criteria which was last revised in 2018 to prevent gift authorship.^6^.^7^
    - Make sure number of authors should be less especially when you are a novice author as it will produce good impression on the editor.
    - Clearly define roles of all authors according to ICMJE criteria of authorship and take signatures from all (manual/ electronic).
    - All authors should declare their contribution towards manuscript writing and final approval and make themselves accountable for integrity of their research.
  - *Ethics Committee / IRB approval*.
  - *Evidence of processing fee submission (non-refundable)*.
  - *Any other supplementary file* (Questionnaire, Transcript, Analysis etc.).
8. You know the quality of your manuscript either it has some important message, does it add some new information to the medical literature or if there are chances of its further citations, only then consider submitting it to an Impact Factor Journal otherwise select some other good quality journal to avoid disappointment of rejection from an IF Journal. In case of submission to IF journal, make sure to visit their website and if possible, add few relevant references from there which will further increase the chances of your manuscript being accepted because the Editors of IF Journals are always conscious and keen to improve their existing Impact Factor.
9. Make sure that you highlight the strengths of your study and also include Limitations of your study under a separate sub-heading as a part of the discussion in the last paragraph.
10. Upload your manuscript along with all the supplementary files carefully. Upload the structured abstract and key words in Meta data on the journal’s website and make sure while uploading the manuscript text, it should be in one file starting from title page till conclusions. Title page includes names and affiliation of all authors, e-mail and contact number of corresponding author, total word count, total number of images and tables and a short running title within footer. Usually at initial screening stage, majority of the manuscripts are not accepted for further processing and the journal might convey the deficiencies to the corresponding author after initial screening which is also known as internal review.^1^ In such a case, make up the deficiencies and resubmit the manuscript along with a covering note how you have responded point by point and highlight all the changes you have made.
11. Please wait at least two-three months after acceptance at submission stage and then you can humbly ask editor about progress on your manuscript by providing title and ID Number. Do not get frustrated as you are dealing with your manuscript only, but for the editor, there are number of manuscripts waiting to be processed at different stages of publishing process. Therefore, keep waiting and have patience.
12. Always plan for at least six to eight months from submission to publication. Standard Impact Factor Journals may take from six months to over a year from the submission till final acceptance and publication. Keep in mind that initial screening, plagiarism check, formatting and then uploading this formatted manuscript on the website takes couple of weeks as there is no short cut.
13. When you are asked to revise the manuscript in the light of reviewer’s comments received from the journal, respond to each and every comment carefully. Be honest in case you cannot respond to any of the point or you do not agree with the reviewer’s comments, politely convey your views to the Editor and also give reason or reference if any. Honesty is appreciated by the Editors. Highlight all the changes and additions made to the initial manuscript to ease Editor’s task in making quick decision, otherwise the revised manuscript will have to be sent to the reviewers again and it will take additional time.
14. Make sure all the listed authors have read and approved the revised manuscript before resubmitting with the covering note stating how you have addressed the reviewer’s comments point by point.
15. After acceptance, editor will send you the pdf file for proof reading. Convey the corrections immediately if any, so that it can be processed further and finally gets published. This is a routine process for most of the standard journals.
16. The manuscript after corrections may be published on journal’s website ahead of print. Some journals who do have acquired DOI number, generate and allot these numbers before ahead of print publication. So, the manuscript will have DOI number only without page numbers. Have a look to ensure that the corrections you already conveyed have been carried out and if few are missed, there is still time to get it corrected. Point it out immediately to the Editor and request for doing the needful.
17. Once all the manuscripts scheduled to appear in a particular issue are finalized, then page numbering is done and pdf files are made once again and then published. This process may take two to three days and during this period your earlier published manuscript may disappear from the journal’s website since new pdf files are under process.
18. Those journals who are covered by PubMed Central and are visible on PubMed, Medline through PubMed Central, while going ahead with the pint copies also arrange to get the XML files made. This software and facility is unfortunately not available in Pakistan so far hence, they get it prepared from overseas and after checking, it is then uploaded on PubMed and this process takes about four to five weeks before these manuscripts are accessible on the Medline. Be patient and do not send e-mails to the Editor inquiring about visibility of your manuscripts on Medline which will only irritate the Editor.

**HAPPY PUBLISHING NOVICE AUTHOR**
